# Supplementary material for: Deconvolution of cancer cell states by the XDec-SM method
Source: PLoS Comput Biol. 2023 Aug 14;19(8):e1011365. doi: 10.1371/journal.pcbi.1011365 (PMC10449115; doi:10.1371/journal.pcbi.1011365)
Supplement: S1 Fig — (A) Heatmap representing the transformed gene expression counts of the 274 informative genes across the pseudo bulk reference expression profiles generated from the scRNA-seq gene expression profiles. (B) Heatmap representing the correlation between the XDec-SM estimated expression profiles (n = 9) and the pseudo bulk reference expression profiles. Red boxes are placed over the highest correlation. XDec-SM estimates five epithelial profiles, two stromal profiles, one T cell profile, and one macrophage profile. (C) Heatmap representing the per-sample proportion of the nine constituent cell types in the TCGA BRCA dataset. Top color bar represents the PAM50 expression subtypes. Only samples with identified subtypes are included in the heatmap. (D) Boxplot representing the per-sample proportion of the nine constituent cell types in the TCGA BRCA dataset separated by the PAM50 classification subtypes. (PDF) [file pcbi.1011365.s001.pdf]

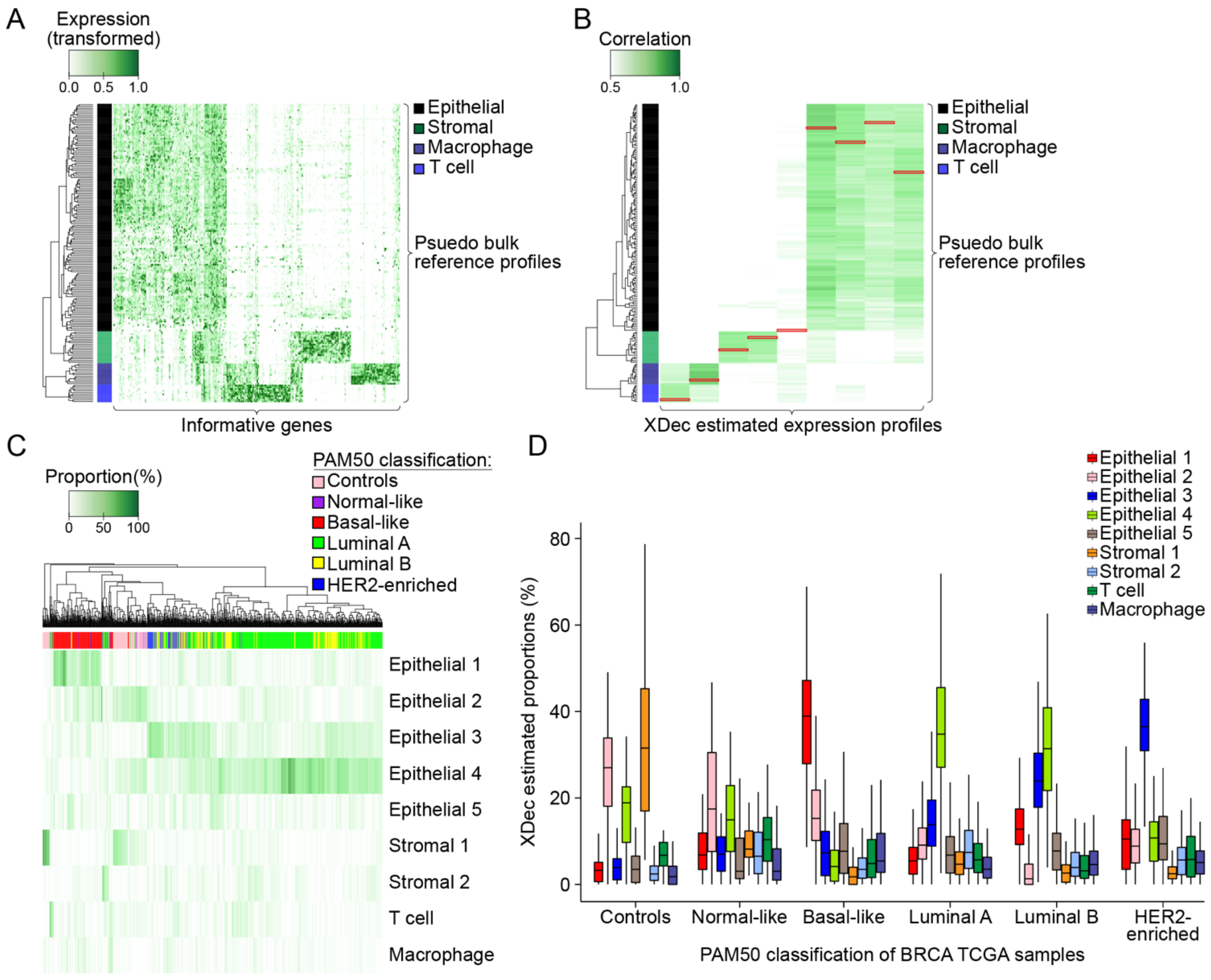

S1 Fig. XDec-SM Deconvolution of RNA-seq profiles of TCGA breast tumor samples utilizing scRNA-seq references

(A) Heatmap representing the transformed gene expression counts of the 274 informative genes across the pseudo bulk reference expression profiles generated from the scRNA-seq gene expression profiles.

(B) Heatmap representing the correlation between the XDec-SM estimated expression profiles (n = 9) and the pseudo bulk reference expression profiles. Red boxes are placed over the highest correlation. XDec-SM estimates five epithelial profiles, two stromal profiles, one T cell profile, and one macrophage profile.

(C) Heatmap representing the per-sample proportion of the nine constituent cell types in the TCGA BRCA dataset. Top color bar represents the PAM50 expression subtypes. Only samples with identified subtypes are included in the heatmap.

(D) Boxplot representing the per-sample proportion of the nine constituent cell types in the TCGA BRCA dataset separated by the PAM50 classification subtypes.
