# Supplementary material for: Deconvolution of cancer cell states by the XDec-SM method
Source: PLoS Comput Biol. 2023 Aug 14;19(8):e1011365. doi: 10.1371/journal.pcbi.1011365 (PMC10449115; doi:10.1371/journal.pcbi.1011365)
Supplement: S2 Fig — The cell type specific gene expression of marker genes across different breast cancer subtypes. ESR1 and FOXA1 are most highly expressed in the epithelial compartment. ADIPOQ and FABP4 are most highly expressed in the stromal adipocyte compartment. FN1 and COL1A1 are most highly expressed in the stromal CAF compartment. CD3G and CD8A are most highly expressed in the immune compartment. (PDF) [file pcbi.1011365.s002.pdf]

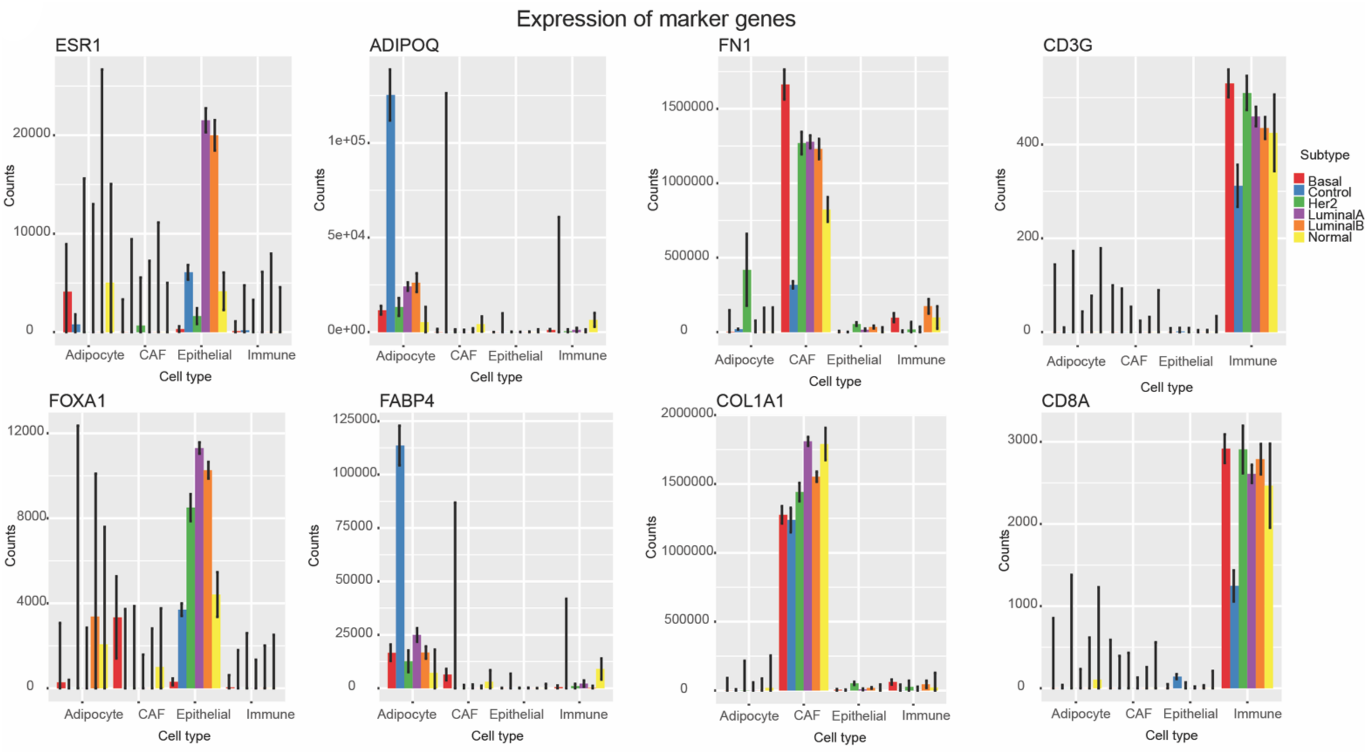

S2 Fig. Cell type specific gene expression

(A) The cell type specific gene expression of marker genes across different breastcancer subtypes. ESR1 and FOXA1 are most highly expressed in the epithelial compartment. ADIPOQ and FABP4 are most highly expressed in the stromal adipocyte compartment. FN1 and COL1A1 are most highly expressed in the stromal CAF compartment. CD3G and CD8A are most highly expressed in the immune compartment.
