# Supplementary material for: Deconvolution of cancer cell states by the XDec-SM method
Source: PLoS Comput Biol. 2023 Aug 14;19(8):e1011365. doi: 10.1371/journal.pcbi.1011365 (PMC10449115; doi:10.1371/journal.pcbi.1011365)
Supplement: S3 Fig — (A-H) Single cell RNA-seq and bulk RNA seq for the same tumor plotted on the cancer cell state map. to visualize the single cell and pooled data for this sample. The pooled sample is composed of ~1X105 cells, while the single cell samples are individual cells. (PDF) [file pcbi.1011365.s003.pdf]

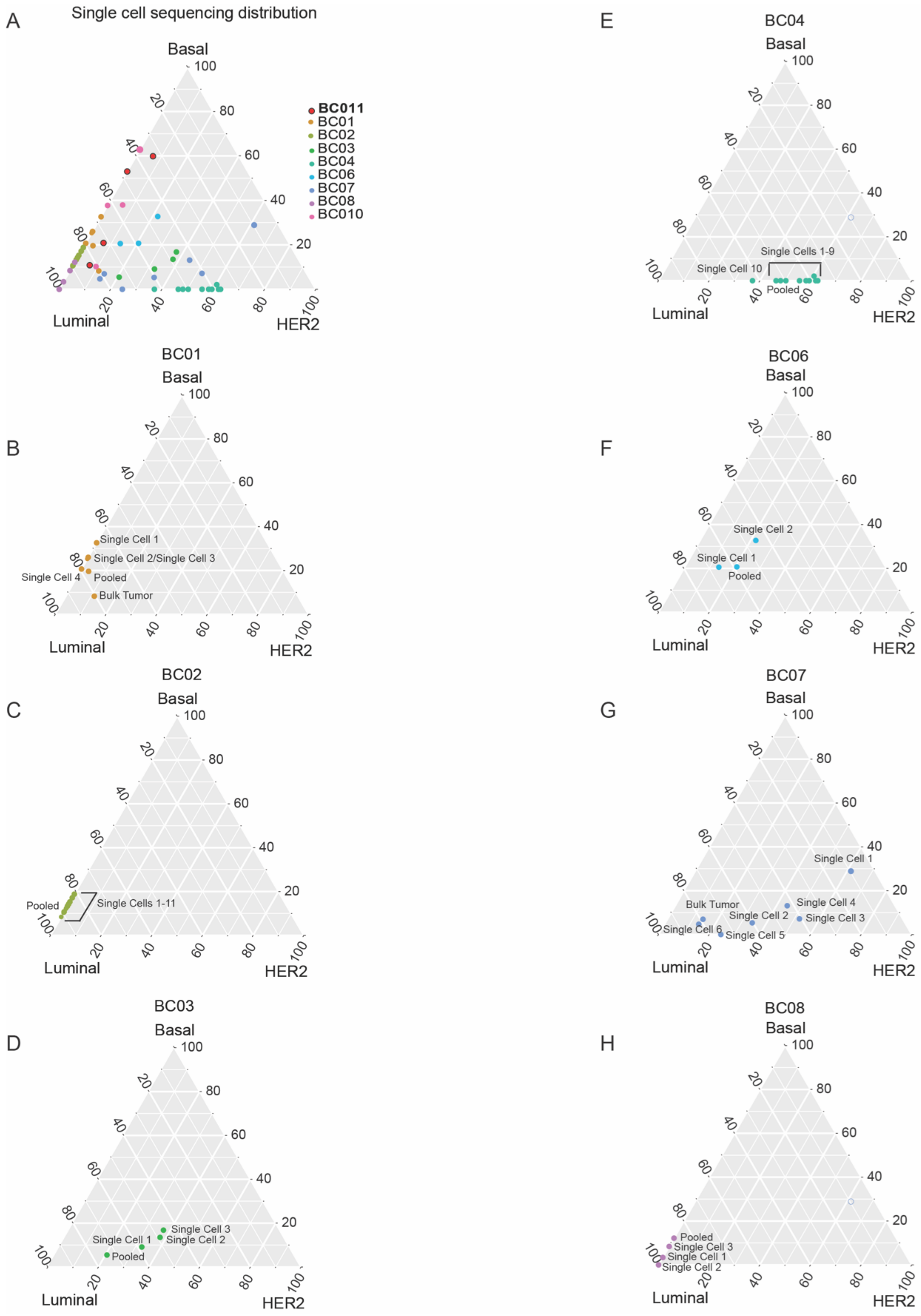

S3 Fig. Distribution of scRNA-seq profiles for individual tumor

(A) Visualization of deconvoluted single cell and bulk expression profiles for 9 unique tumors. Dots of the same color correspond to samples derived from the same tumor.
(B-H) Single cell RNA-seq and bulk RNA seq for the same tumor plotted on the cancer cell state map.
