# Supplementary material for: Deconvolution of cancer cell states by the XDec-SM method
Source: PLoS Comput Biol. 2023 Aug 14;19(8):e1011365. doi: 10.1371/journal.pcbi.1011365 (PMC10449115; doi:10.1371/journal.pcbi.1011365)
Supplement: S4 Fig — (A) Survival curve showing no significant survival difference between patients classified as Luminal by PAM50 and HER2 negative (blue) and Luminal by PAM50 and HER2 positive (pink). (B) Survival curve showing no significant survival difference between patients classified as Luminal by PAM50 and with high ERBB2 pathway activation (blue) and Luminal by PAM50 and without high ERBB2 pathway activation (pink). (PDF) [file pcbi.1011365.s004.pdf]

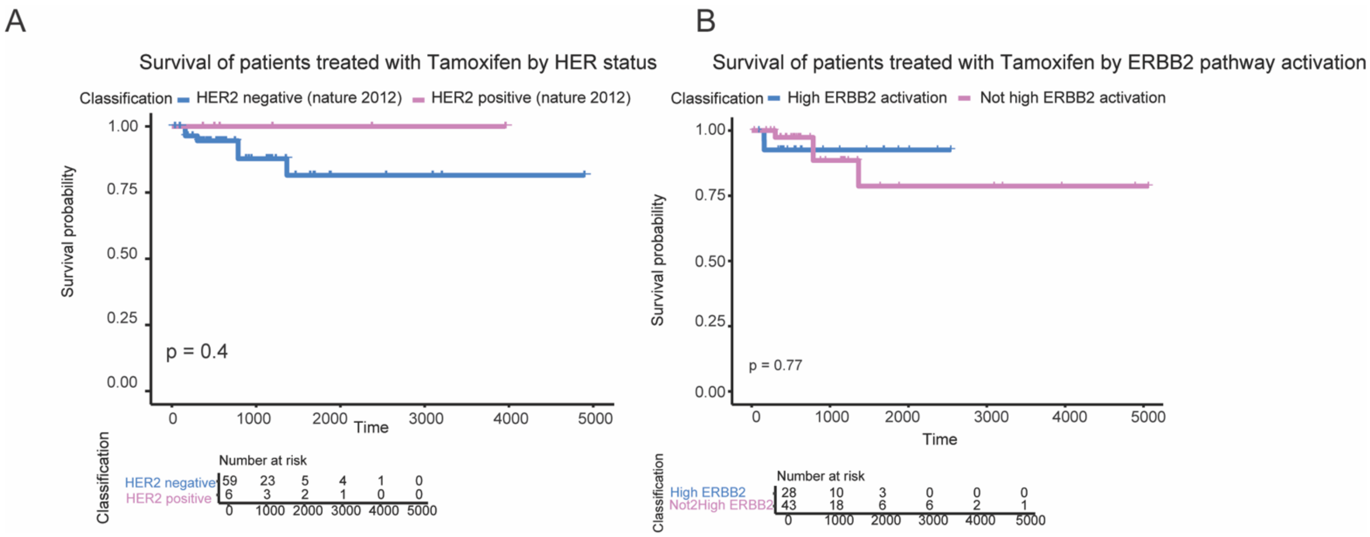

S4 Fig. Survival curves for patients treated with Tamoxifen

(A left) Survival curve showing no significant survival difference between patients classified as Luminal by PAM50 and HER2 negative (blue) and Luminal by PAM50 and HER2 positive (pink). (A right) Survival curve showing no significant survival difference between patients classified as Luminal by PAM50 and with high ERBB2 pathway activation (blue) and Luminal by PAM50 and without high ERBB2 pathway activation (pink).
